# N-Acetylcysteine breaks resistance to trastuzumab caused by MUC4 overexpression in human HER2 positive BC-bearing nude mice monitored by ^89^Zr-Trastuzumab and ^18^F-FDG PET imaging

**DOI:** 10.18632/oncotarget.17015

**Published:** 2017-04-10

**Authors:** Zéna Wimana, Geraldine Gebhart, Thomas Guiot, Bruno Vanderlinden, Denis Larsimont, Gilles Doumont, Gaetan Van Simaeys, Serge Goldman, Patrick Flamen, Ghanem Ghanem

**Affiliations:** ^1^ Nuclear Medicine Department, Institut Jules Bordet, Université Libre de Bruxelles, Brussels, Belgium; ^2^ Laboratory of Oncology and Experimental Surgery, Institut Jules Bordet, Université Libre de Bruxelles, Brussels, Belgium; ^3^ Pathology Department, Institut Jules Bordet, Université Libre de Bruxelles, Brussels, Belgium; ^4^ Center for Microscopy and Molecular Imaging (CMMI), Université Libre de Bruxelles, Brussels, Belgium

**Keywords:** HER2, NAC, trastuzumab, resistance, immunoPET

## Abstract

Trastuzumab remains an important drug in the management of human epidermal growth factor receptor 2 (HER2) overexpressing breast cancer (BC). Several studies reported resistance mechanisms to trastuzumab, including impaired HER2-accessibility caused by mucin 4 (MUC4). Previously, we demonstrated an increase of Zirconium-89-radiolabeled-trastuzumab (^89^Zr-Trastuzumab) accumulation when MUC4-overexpressing BC-cells were challenged with the mucolytic drug N-Acetylcysteine (NAC). Hereby, using the same approach we investigated whether tumor exposure to NAC would also enhance trastuzumab-efficacy.

Dual SKBr3 (HER2+/MUC4-, sensitive to trastuzumab) and JIMT1 (HER2+/MUC4+, resistant to trastuzumab) HER2-BC-bearing-xenografts were treated with trastuzumab and NAC. Treatment was monitored by molecular imaging evaluating HER2-accessibility/activity (^89^Zr-Trastuzumab HER2-immunoPET) and glucose metabolism (^18^F-FDG-PET/CT), as well as tumor volume and the expression of key proteins.

In the MUC4-positive JIMT1-tumors, the NAC-trastuzumab combination resulted in improved tumor-growth control compared to trastuzumab alone; with smaller tumor volume/weight, lower 18F-FDG uptake, lower %Ki67 and pAkt-expression. NAC reduced MUC4-expression, but did not affect HER2-expression or the trastuzumab-sensitivity of the MUC4-negative SKBr3-tumors.

These findings suggest that improving HER2-accessibility by reducing MUC4-masking with the mucolytic drug NAC, results in a higher anti-tumor effect of trastuzumab. This provides a rationale for the potential benefit of this approach to possibly treat a subset of HER2-positive BC overexpressing MUC4.

## INTRODUCTION

The Human epidermal growth factor receptor 2 (HER2) transmembrane oncoprotein is overexpressed in 20–30% of breast cancer (BC) patients [1, 2]. HER2 overexpression in BC has been associated with an aggressive biological behavior, translated into shorter disease-free interval and overall survival in patients with early and advanced disease states [3, 4]. Trastuzumab, a recombinant, humanized monoclonal antibody (mAb) was the first clinically approved anti-HER2 therapy. It specifically binds to HER2 on the C-terminal portion of the extracellular domain (ECD) near the juxtamembrane region in domain IV of the HER2 receptor.

The proposed mechanisms of action of trastuzumab are multiple. It's most well-known and crucial mechanism of action is the inhibition of the PI3K/Akt pathways. Trastuzumab interferes with HER2 activation and so results in the suppression of Akt phosphorylation [5, 6].

Trastuzumab used in addition to standard chemotherapy has been shown to improve treatment outcome of early as well as metastatic stage in HER2-positive BC [7, 8]. However, despite this success, responses to trastuzumab have been hampered by several resistances mechanisms [9, 10]. One of these is the overexpression of high molecular weight membrane-anchored mucin MUC4 [9].

MUC4 is overexpressed in 30–95% of all types of BC as well as lymph nodes metastases and tumor vascular emboli. MUC4 has been correlated with progression and higher tumor grade [11–13]. This large glycoprotein can considerably hinder the accessibility and hence the binding of trastuzumab to HER2 ectodomain, thereby impairing efficient trastuzumab-based treatment [14, 15]. MUC4 has also been postulated to interact closely with HER2 to form a ligand-receptor type intramembrane complex. Herein, it has been shown to operate as a ligand/modulator of HER2 activation by inducing the phosphorylation of HER2 on tyr1248 [12, 16–19].

In a previous molecular imaging study, HER2 receptor impaired accessibility by MUC4 in BC was shown to be appropriately assessed by *in vivo* molecular imaging using PET and Zirconium-89 radiolabeled trastuzumab (^89^Zr-Trastuzumab). Importantly, mucolytic treatment N-Acetylcysteine (NAC) restored the impaired HER2 accessibility caused by MUC4, and consequently enhanced the binding and uptake of ^89^Zr-Trastuzumab *in vitro* and *in vivo* in a MUC4-expressing HER2-positive BC xenograft mouse model [19].

NAC is known to be a safe, well-tolerated, well-documented and inexpensive drug [18, 19]. Furthermore, it is more effective than any other mucolytic drug and has therefore been the most widely used [20]. NAC presents an acetyl group promoting its binding to the cell membrane and a free thiol group able to reduce intra- or intermolecule disulfide bonds between cysteine residues of mucins [18, 20], leading to reduced masking of HER2 [19]. Of, note NAC has also been reported to indirectly inhibit mTOR, downstream of the PI3K/akt pathway [23].

**Figure 1:**
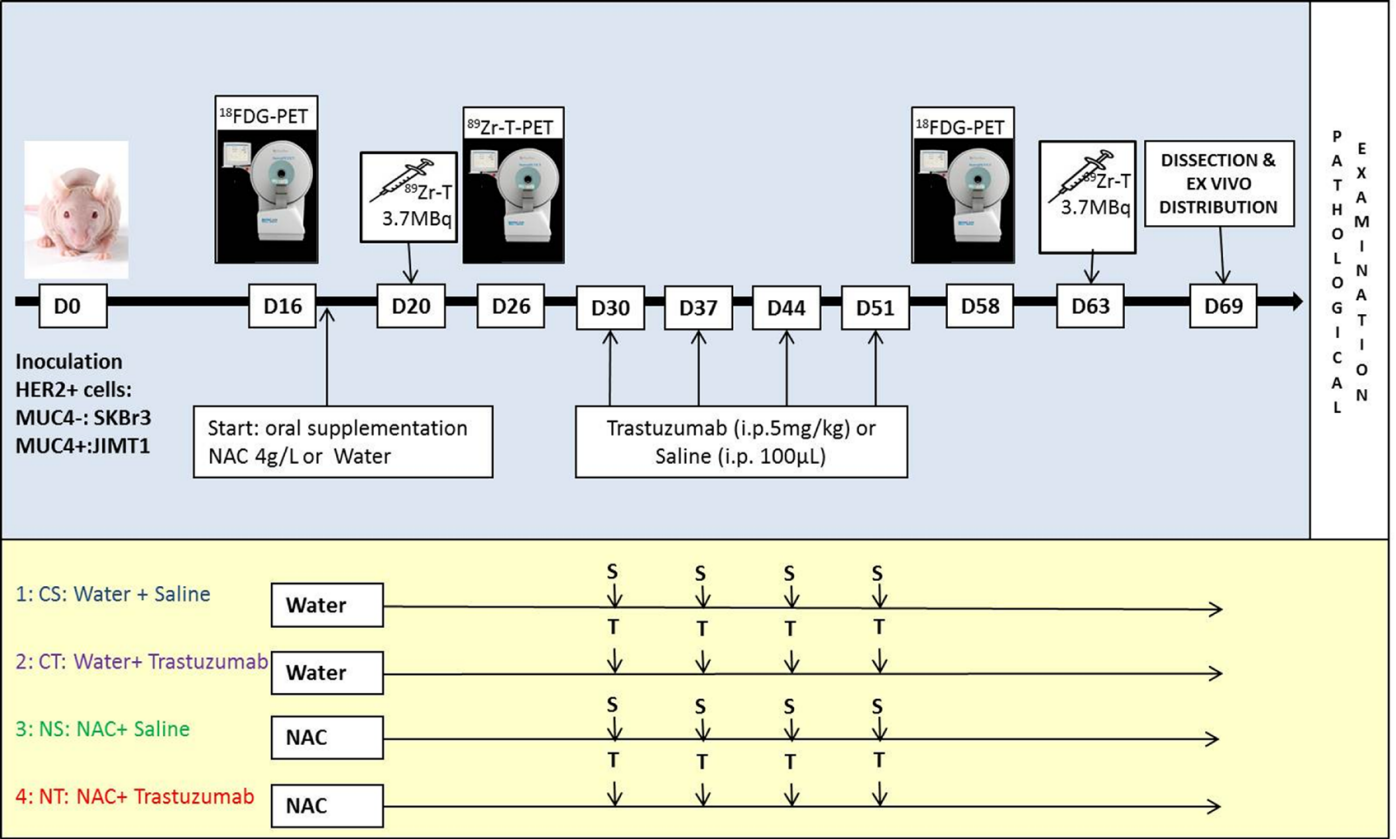
Timeline of the animal experimentation Athymic nu/nu female mice were inoculated subcutaneously on the right posterior leg with SKBr3 (HER2+/MUC4-) cells and on the left with JIMT1 (HER2+/MUC4+) cells. Imaging and treatments were initiated two weeks after inoculation when tumors reached approximately 0.5 cm^3^. Mice were randomized to four different groups: (1) the CS group receiving sweetened drinking water and i.p. saline injections (*n* = 10), (2) the CT group receiving sweetened drinking water and trastuzumab injections (i.p., 5mg/kg (*n* = 10), (3) the NS group receiving sweetened drinking water supplemented with NAC and saline i.p. injections (*n* = 9), and (4) the NT group receiving sweetened drinking water supplemented with NAC and trastuzumab injections (i.p., 5mg/kg) (*n* = 10). Imaging studies were conducted using a μPET-CT and each mouse was scanned three times, with an ^18^F-FDG and ^89^Zr-Trastuzumab PET-CT before treatment and an ^18^F-FDG PET-CT post treatment. Images were recorded for ^18^F-FDG at 1h and ^89^Zr-Trastuzumab 6 days after injection respectively. After the last scan, animals were euthanized and an ex-vivo body distribution study was performed. A pathological examination was also conducted on a subset of animals from each cohort with immunostaining of HER2, MUC4, pAkt and Ki67.

In the present study, we hypothesized that MUC4-related resistance to trastuzumab can be overcome through the use of a mucolytic drug, by improving the accessibility of the drug to HER2 and hence achieve a better anti-tumor effect. This approach was evaluated in a dual JIMT1 (HER2+/MUC4+; resistant to trastuzumab) and SKBr3 (HER2+/MUC4-; control sensitive to trastuzumab) HER2 human BC-bearing xenograft model by molecular imaging techniques ^89^Zr-Trastuzumab HER2-immunoPET and ^18^F-FDG PET-CT; as well as measurement of tumor volume and pathological examination (Figure 1).

## RESULTS

### Mucolytic drug NAC enhances HER2 accessibility for trastuzumab in MUC4-overexpressing tumors as shown on ^89^Zr-Trastuzumab immunoPET

**Figure 2:**
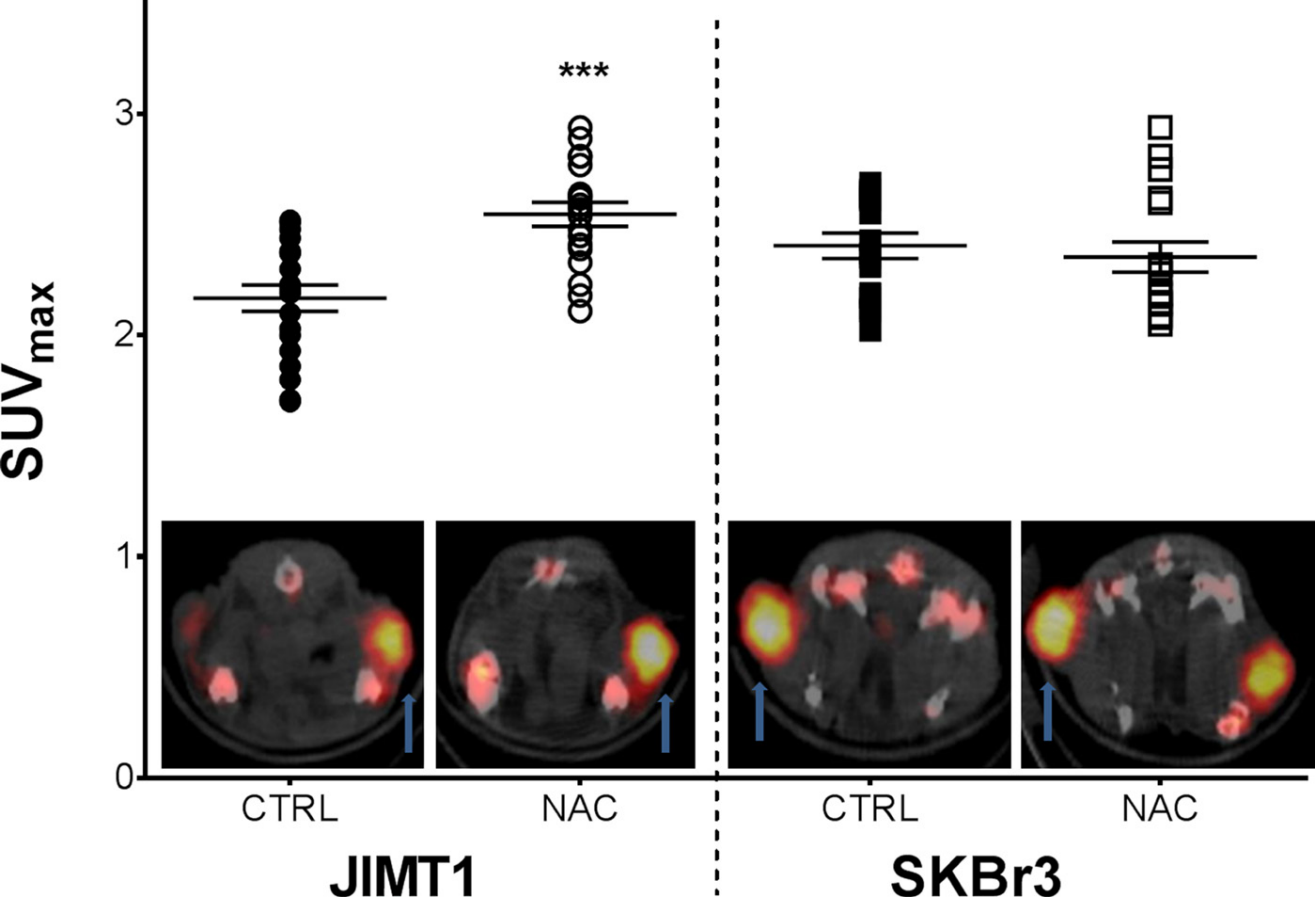
Mucolytic drug NAC enhances ^89^Zr-Trastuzumab uptake in MUC4 overexpressing JIMT1 tumors Standardized maximum uptake of ^89^Zr-Trastuzumab in JIMT1 (HER2+/MUC4+; dot) and SKBr3 (HER2+/MUC4-; squares) tumors under NAC supplementation (*n* = 19) and control (*n* = 20) are shown in the graph with a ^89^Zr-Trastuzumab PET axial image representative of the tumor (arrow) uptake under NAC exposure (NAC) and control (CTRL). All data points and mean ± SEM are shown, both expressed in SUV_max_ with ***p < 0.001.

Baseline ^89^Zr-Trastuzumab PET imaging at 6 days post injection revealed tumor uptake in all mice in both NAC and control group. ^89^Zr-Trastuzumab also showed visible uptake in the liver and bones. Image analysis demonstrated a statistically significant increase in radiotracer uptake, represented by %ΔSUV_max_ (33.7 ± 7.4%, *p* < 0.001) of MUC4 overexpressing JIMT1 tumors in mice treated with NAC (2.55 ± 0.05) compared to untreated controls (2.17 ± 0.06). In contrast, no significant differences were observed in ^89^Zr-Trastuzumab uptake for MUC4-negative SKBr3 tumors implanted in the same animal (NAC: 2.40 ± 0.06 vs. controls: 2.35 ± 0.07) (Figure 2).

### NAC supplementation improves the antitumor activity of trastuzumab in MUC4-overexpressing tumors

#### Tumor volume monitoring

**Figure 3:**
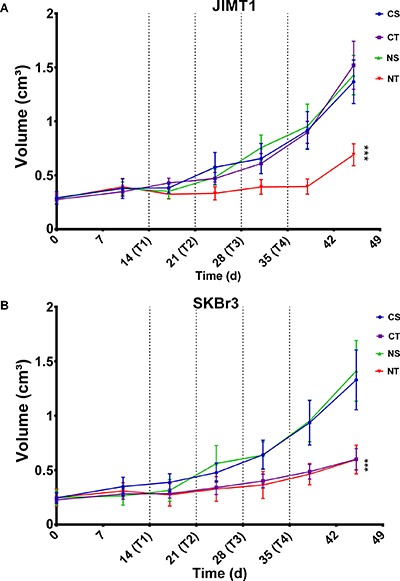
The combination of trastuzumab with NAC supplementation results in slower tumor growth The effect of the different treatment on JIMT1 (HER2+/MUC4+) and SKBr3 (HER2+/MUC4) tumors in dual-tumor-bearing mice randomized to the four treatment arms CS (Control+Saline; blue, *n* = 10), CT (Control+Trastuzumab; purple, *n* = 10), NS (NAC+Saline; green, *n* = 9) and NT (NAC+Trastuzumab; red *n* = 10) is represented per methodologies as follows: Tumor growth of JIMT1 (**A**) and SKBr3 (**B**) tumors monitored through caliper measurements. Data are represented as mean ± SEM, expressed in cm^3^ for tumor volume, with ***p < 0.001.

Longitudinal assessment of tumor growth revealed an increase in subcutaneous tumor volume in all tumor types and all treatment arms with different tumor-growth-rates (Figure 3A and 3B). At the end of the treatment period and in the trastuzumab-sensitive SKBr3 tumors, trastuzumab significantly induced slower cell doubling times compared to untreated controls, with or without NAC (CT=32.3 days vs CS=20.3 and NT=30.1 vs NS=20.8, all *p* < 0.001) and consequently smaller tumor volumes (CT= 0.60 ± 0.10 cm^3^ vs CS=1.33 ± 0.27, NT=0.60 ± 0.13 vs NS=1.41 ± 0.27, both *p* < 0.001). In contrast, in the trastuzumab-resistant JIMT1, inhibition of tumor growth was only obtained when trastuzumab was combined with NAC (NT = 33.9 days vs CT = 21.0, NS = 21.7 and CS=21.6, all *p* < 0.001) resulting in smaller tumor volume in the NT group (NT=0.69 ± 0.10 cm^3^ vs CT = 1.52 ± 0.22, NS = 1.43 ± 0.18 and CS = 1.37 ± 0.20, all *p* < 0.001).

These results were corroborated by *in vivo* measurements on CT as well as confirmed by tumor weight at dissection (Supplementary Figure 1).

### ^18^F-FDG PET-CT assessment

After treatment completion, the effects of trastuzumab treatment on tumor metabolism were assessed through changes in ^18^F-FDG uptake (%ΔSUV_max_).

**Figure 4:**
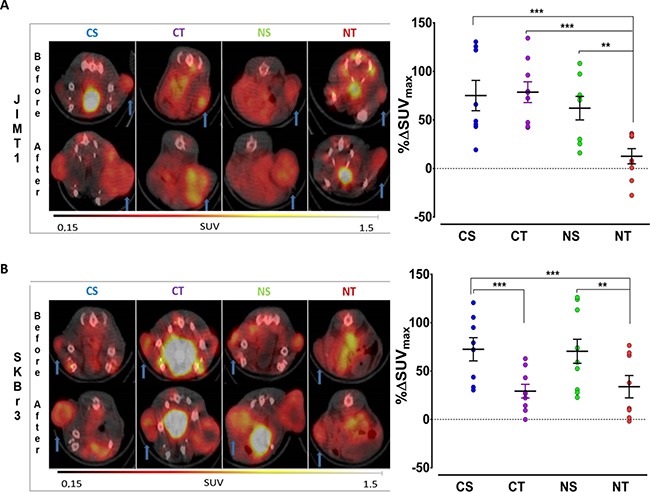
The combination of trastuzumab with NAC supplementation results in a lower ^18^F-FDG uptake in trastuzumab resistant tumors ^18^F-FDG uptake of SKBr3 (HER2+/MUC4-) and JIMT1 (HER2+/MUC4+) tumors in dual-tumor-bearing mice randomized to the four treatment arms CS (Control+Saline; blue, *n* = 10), CT (Control+Trastuzumab; purple, *n* = 10), NS (NAC+Saline; green, *n* = 9) and NT (NAC+Trastuzumab; red *n* = 10) is represented. (**A**) The baseline ^18^F-FDG uptake of JIMT1 and SKBr3 tumors preceding randomization is shown with future treatment group allocation. Representative ^18^F-FDG PET/CT axial images(left) of the tumor (arrow) before and after treatment are shown together with the changes in ^18^F-FDG uptake represented as percentage difference in SUV_max_ (%*ΔSUV*_max_, right) for (**B**) JIMT1 and SKBr3 tumors in the different treatment arms. Data points as well as mean ± SEM are shown. Data are expressed in SUV_max_ for the baseline ^18^F-FDG uptake or as the percentage difference in SUV_max_ (%*ΔSUV*_max_) when comparing the effect of the different treatment arms; with **p < 0.01 and ***p < 0.001.

In the MUC4-overexpressing trastuzumab resistant JIMT1 tumors, tumor metabolic changes were observed exclusively in the treatment arm combining trastuzumab and NAC with a significantly lower %ΔSUV_max_ (NT = 12.6 ± 7.8 % vs CT = 78.6 ± 10.6, NS = 62.1 ± 12.1 and CS = 75.1 ± 15.5; all *p <* 0.01) (Figure 4B). Whereas in the control SKBr3 tumors, trastuzumab-based treatment led to a significant reduction of tumor activity compared to the respective controls and this independent of NAC supplementation (%ΔSUV_max_: CT = 29.2 ± 7.1 % vs CS = 72.5 ± 12.5and NT = 33.8 ± 11.6 vs NS = 75.7 ± 12.6 %; *p* < 0.001 and *p* < 0.01) (Figure 4C). Supplementation with NAC alone did not induce any effect on tumor metabolic changes in either tumor types compared to controls.

### Proliferation index Ki67 and activation of the Pi3K/Akt pathway

**Figure 5:**
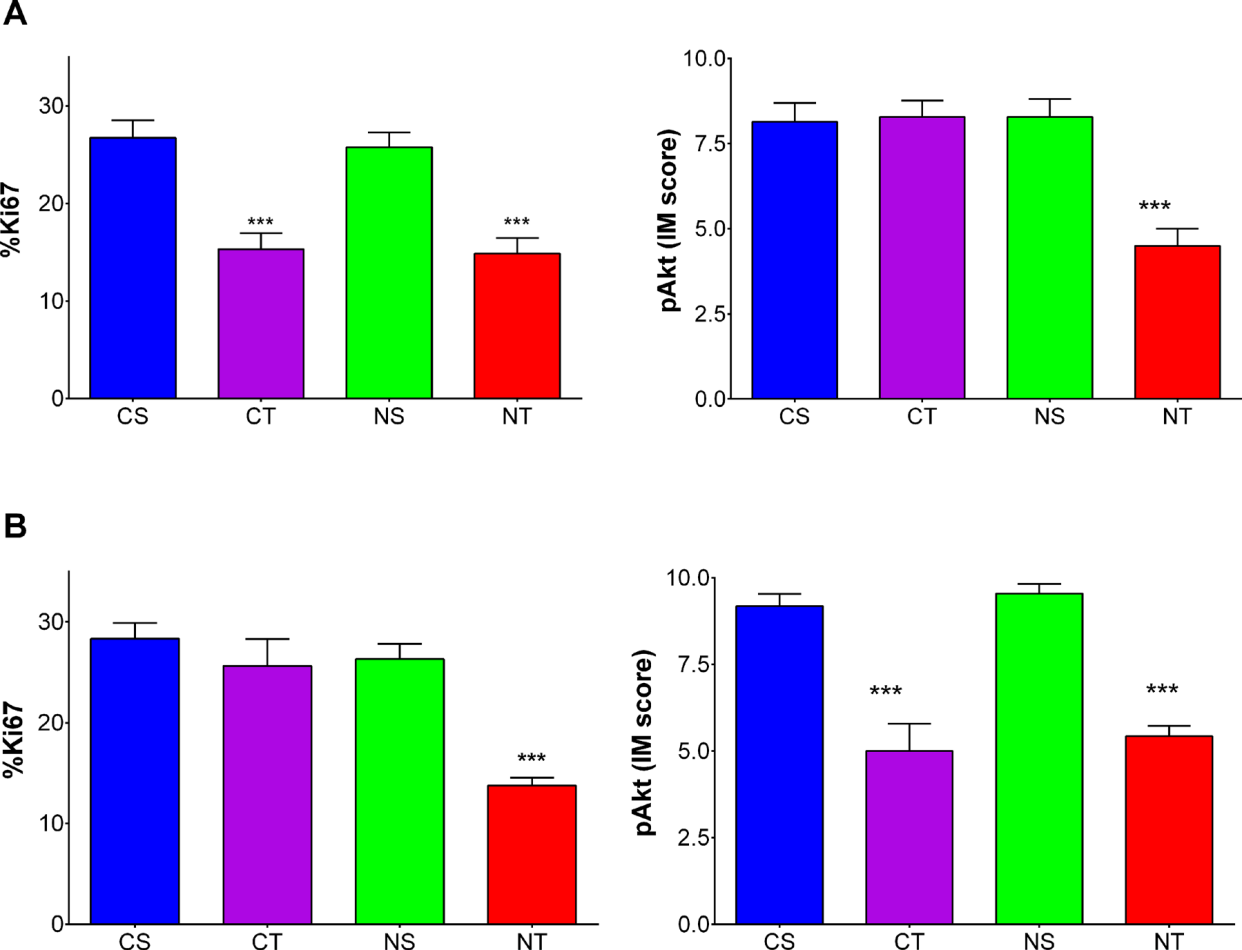
Combining trastuzumab with NAC supplementation reduces proliferation and activation of the molecular Pi3K/Akt signaling pathway in trastuzumab resistant tumors proliferation index Ki67 (left) and pAkt levels (right) for (**A**) JIMT1 (HER2+/MUC4+) and (**B**) SKBr3 (HER2+/MUC4-) tumors in dual-tumor-bearing mice randomized to the four treatment arms CS (Control+Saline; blue, *n* = 5), CT (Control+Trastuzumab; purple, *n* = 5), NS (NAC+Saline; green, *n* = 5) and NT (NAC+Trastuzumab; red *n* = 5). Data are shown as mean ± SEM expressed in % positive cells for Ki67 index values and IM scores for pAkt levels with ***p < 0.001.

In the MUC4-overexpressing trastuzumab resistant JIMT1 tumors, the percentage of Ki67 positive cells was decreased only in the NAC-trastuzumab combination arm compared to the other groups (NT=13.8 ± 0.8 % vs CT=25.6 ± 2.7, NS=26.3 ± 1.5 and CS=28.3 ± 1.6; *p* < 0.001) (Figure 5). The pAkt expression levels of those JIMT1 tumors in the NT combination arm were lower as well (NT=4.5 ± 0.5 vs CT=8.3 ± 0.5, NS=8.3 ± 0.5 and CS=8.2 ± 0.6; *p* < 0.001) (Figure 5A). In the control SKBr3 tumors, trastuzumab treatment, with or without NAC supplementation, resulted in a decrease in Ki67 positive tumor cells compared to the respective controls (CT = 15.3 ± 1.6 % vs CS = 26.7 ± 1.8 and NT = 14.9 ± 1.6 vs NS=25.8 ± 1.5; *p* < 0.001 and *p* = 0.028) (Figure 5B). Also, the pAkt level were about twice lower in trastuzumab-treated SKBr3 tumors compared to the control group (CT = 5.0 ± 0.8 vs CS = 9.2 ± 0.4 and NT = 5.4 ± 0.3 vs NS = 9.5 ± 0.3; both *p* < 0.001) (Figure 5B). Representative samples of the pathological examination of the Ki67 index and pAkt expression levels are shown in Supplementary Figure 2.

### Prolonged NAC supplementation does not alter ^89^Zr-Trastuzumab body distribution in tumor-bearing mice

**Figure 6:**
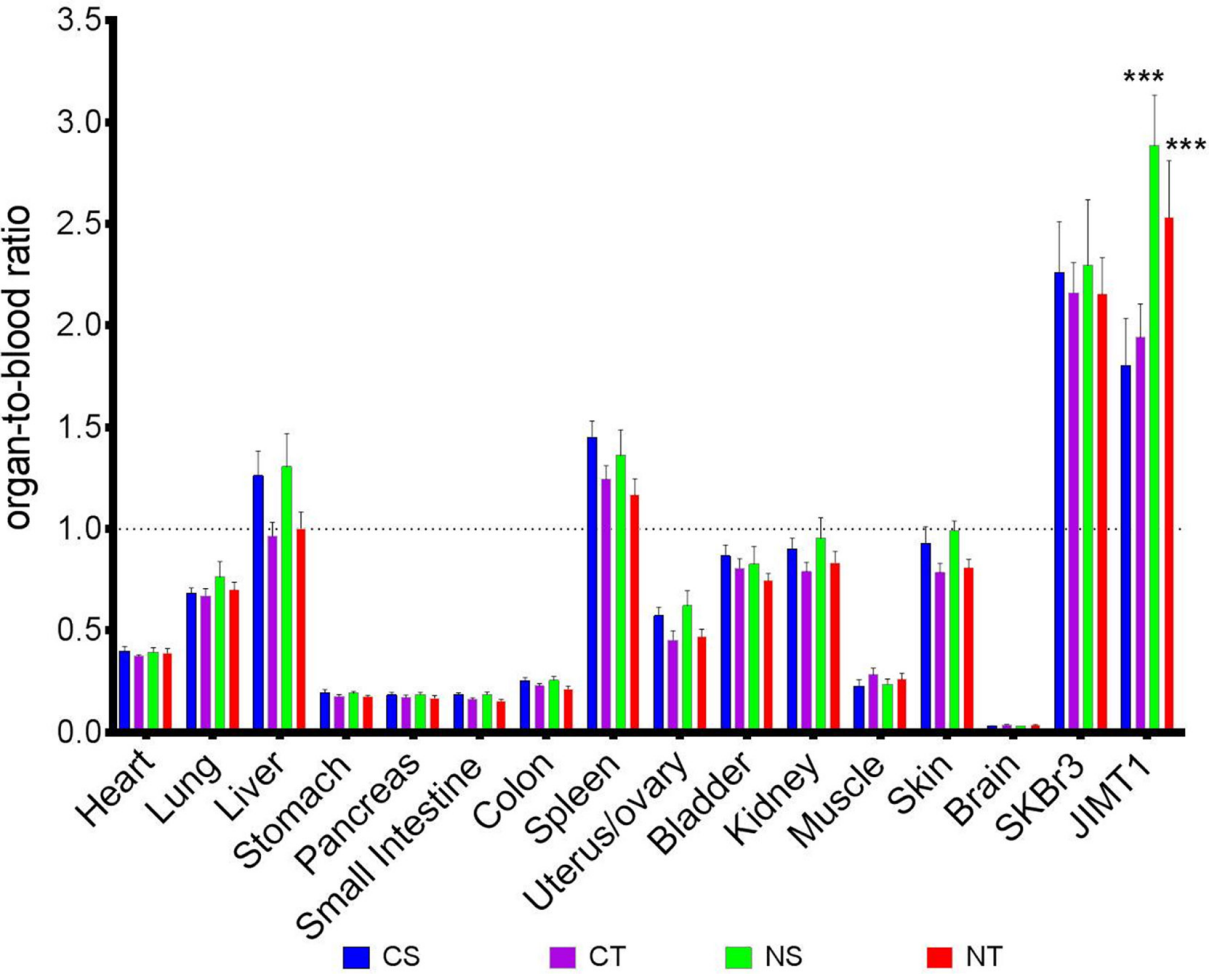
Prolonged NAC supplementation does not alter ^89^Zr-Trastuzumab body distribution, with the exception of the maintained enhanced uptake in MUC4-overexpressing tumors *Ex vivo* body distribution of ^89^Zr-Trastuzumab uptake in JIMT1 (HER2+/MUC4+) and SKBr3 (HER2+/MUC4-) tumors in dual-tumor-bearing mice randomized to the four treatment arms CS (Control+Saline; blue, *n* = 10), CT (Control+Trastuzumab; purple, *n* = 10), NS (NAC+Saline; green, *n* = 9) and NT (NAC+Trastuzumab; red *n* = 10). Data are expressed in organ-to-blood ratio and represented as mean ± SEM with ***p < 0.001.

To assess the possible impact of prolonged supplementation of NAC on body distribution of ^89^Zr-Trastutumab, organ radioactivity was measured after 60 days on NAC. High uptake occurred in all tumors, liver and spleen and was negligible in the brain as shown in Figure 6. Radioactivity accumulation in non-tumor as well as in the MUC4-negative SKBr3 tumor was not significantly altered by long term NAC exposure.

In the MUC4-positive JIMT1 tumors treated with NAC, a statistically significant higher radioactivity accumulation was observed compared to respective controls (NS = 2.89 ± 0.25 vs CS = 1.81 ± 0.2 and NT = 2.53 ± 0.28 vs CT = 1.78 ± 0.16; *p* < 0.001 for both). Furthermore, treatment with trastuzumab (CT and NT) only resulted in a trend for lower ^89^Zr-Trastuzumab accumulation compared to their corresponding control arm (CS and NS).

### Comparison of HER2 expression, HER2 accessibility for trastuzumab metabolic response assessed by ^18^F-FDG PET

**Figure 7:**
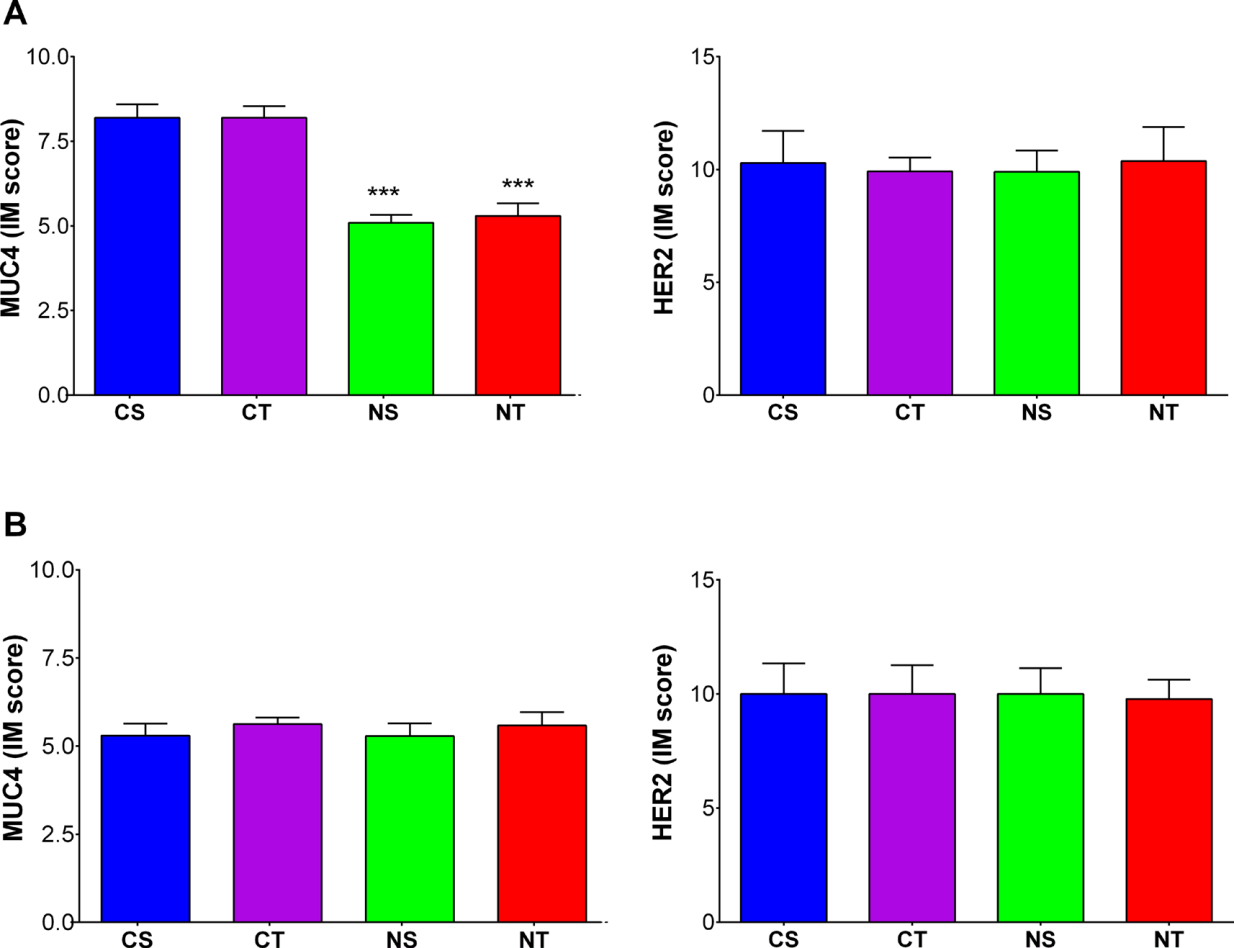
NAC supplementation reduces MUC4 expression in MUC4 overexpressing tumors and HER2 expression is not altered in the different treatment arms MUC4 (left) and HER2 (right) expression levels in (**A**) JIMT1 (HER2+/MUC4+) and (**B**) SKBr3 (HER2+/MUC4-) tumors in dual-tumor-bearing mice randomized to the four treatment arms CS (Control+Saline; blue, *n* = 5), CT (Control+Trastuzumab; purple, *n* = 5), NS (NAC+Saline; green, *n* = 5) and NT (NAC+Trastuzumab; red *n* = 5). Data are shown as mean ± SEM expressed in IM scores with ***p < 0.001.

Immunostaining for MUC4 showed overexpression in JIMT1 tumors, with lower MUC4 expression observed in the groups with NAC supplementation compared to the control groups (NS = 5.1 ± 0.2 vs CS = 8.2 ± 0.4 and NT = 5.3 ± 0.4 vs CT = 8.2 ± 0.3; both *p* < 0.001) (Figure 7A).

On the other hand, heterogeneous immunostaining pattern of intratumor HER2-expression was observed in all tumors. Nonetheless, the mean IM score was similar in all treatment arms (SKBR3: CS = 8.5 ± 1.5, CT = 8.4 ± 1.3, NS=8.3 ± 1.0 and NT = 8.3 ± 0.8; JIMT1: CS=7.3 ± 0.9, CT = 7.3 ± 0.7, NS=7.3 ± 1.1 and NT = 7.3 ± 1.4) (Figure 7B).

**Figure 8:**
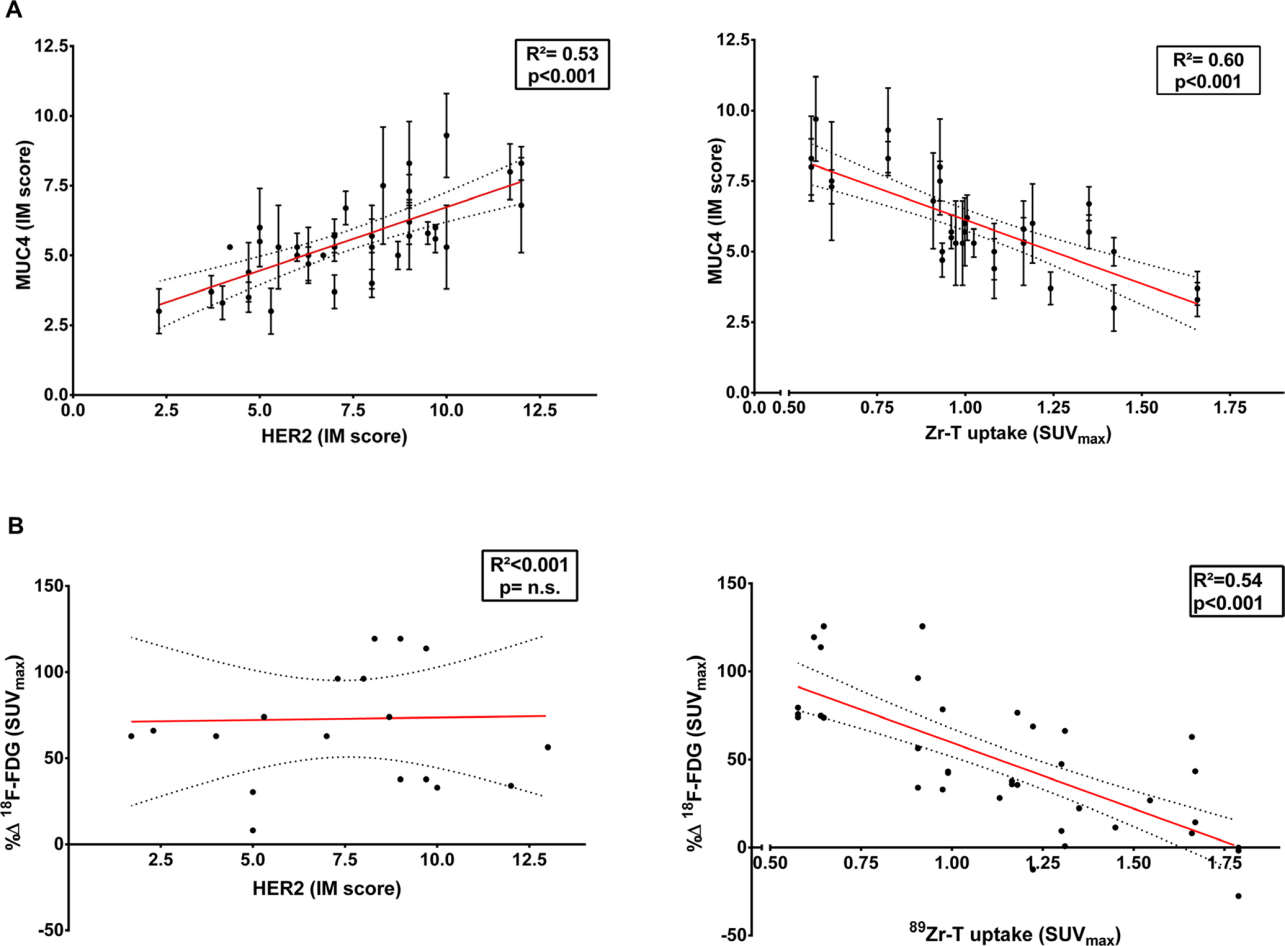
HER2 expression on immunostaining and HER2 accessibility on ^89^Zr-Trastuzumab PET are not concordant in regards to their association with MUC4 or metabolic change on ^18^F-FDG The side by side comparison of the association of HER2 expression levels ( left) and ^89^Zr-Trastuzumab uptake (right) with (**A**) MUC4 is shown including both JIMT1 (HER2+/MUC4+) and SKBr3 (HER2+/MUC4-) tumors in dual-tumor-bearing mice randomized to the four treatment arms CS (Control+Saline), CT (Control+Trastuzumab), NS (NAC+Saline) and NT (NAC+Trastuzumab). (**B**) The side by side comparison of the association to ^18^F-FDG PET metabolic response is limited to mice randomized to the trastuzumab treatment arms CT and NT are also represented. Data shown include both tumor types and are expressed in IM scores for HER2 and MUC4 expression, in SUV_max_ for ^89^Zr-Trastuzumab uptake and expressed as the percentage difference in SUV_max_ (%*ΔSUV*_max_) for ^18^F-FDG metabolic response.

Representative samples of the pathological examination of MUC4 and HER2 exspression levels are shown in Supplementary Figure 2. When comparing expression of MUC4 and HER2, a significant correlation was found between the presence of MUC4 and HER2 (R^2^=0.53; *p* < 0.001). In contrast, an inverse correlation was observed between MUC4 expression and ^89^Zr-Trastuzumab uptake (R^2^ = 0.60; *p* < 0.001) (Figure 8A).

Furthermore, in the trastuzumab-based treatment arms (CT and NT) a significant correlation was found between the metabolic response assessed by ^18^F-FDG PET and HER2 accessibility for trastuzumab through ^89^Zr-Trastuzumab PET (R^2^ = 0.54; *p* < 0.001) but not with HER2 expression (R^2^ < 0.001; *p* = n.s.) (Figure 8B).

## DISCUSSION

This study investigated whether in tumors overexpressing HER2 and MUC4, NAC supplementation could translate into a therapeutic benefit when combined with trastuzumab. Our hypothesis was tested through different methodologies in a dual-BC-xenograft mouse model with human HER2-positive BC tumors, SKBr3 and JIMT1, the latter being resistant to trastuzumab due to MUC4 overexpression. In contrast to the trastuzumab-sensitive SKBr3 tumors, JIMT1 tumors were only responsive when trastuzumab was combined with NAC supplementation. This was demonstrated by (1) an inhibition of tumor growth, (2) smaller tumor volumes as assessed by caliper and CT measurements, (3) a lower ^18^F-FDG accumulation, (4) a smaller Ki67 proliferation index and (5) a lower Akt phosphorylation for mice receiving the NAC-trastuzumab combination.

All these results converge to the same finding that NAC supplementation can significantly break MUC4-related resistance to trastuzumab by restoring HER2 hampered accessibility.

Our previous study demonstrated that for MUC4 overexpressing tumors HER2 hampered accessibility could be improved as a result of NAC administration [19]. Once more, enhanced accessibility of trastuzumab to HER2 was shown with a 30% higher ^89^Zr-Trastuzumab uptake in JIMT1 (HER2+/MUC4+; resistant to trastuzumab) tumors exposed to NAC compared to control mice, without any effect on SKBr3 (HER2+/MUC4-; control sensitive to trastuzumab). Additionally, this study confirmed the effect of NAC on MUC4 specifically, with lower MUC4 expression of JIMT1 tumors in the NAC supplementation groups. Besides consistency with our previously published data, these findings confirm the validity of our model and approach in enhancing the accessibility of HER2 for trastuzumab by decreasing MUC4 related hindrance with mucolytic drug NAC.

In accordance with previously published data [23–25], the SKBr3 tumors that served as control were trastuzumab sensitive and presented about 50% inhibition of tumor growth under trastuzumab treatment irrespective of NAC supplementation. In contrast, for the trastuzumab resistant JIMT1 tumors, a significant anti-proliferative effect was seen only when trastuzumab was combined with NAC with a 60% slower tumor doubling time. Consistent tumor volume results were found with CT measurements showing halved tumor volumes in both trastuzumab treatment groups (with or without NAC) for SKBR3 and only in the trastuzumab-NAC arm for JIMT1 tumors when compared to control. Also, the *ex vivo* measure of tumor weight at dissection validated both results with two times smaller tumors. Trastuzumab did not result in tumor shrinkage/regression but resulted in a change in growth rate. Nevertheless the effect of targeted drugs on the rate of tumor growth have been reported to be more predictive of treatment response rather than tumor shrinkage/regression [27].

Interestingly, the observed overlap at baseline seen in ^89^Zr-Trastuzumab uptake of JIMT1 tumors of mice in the NAC and control group was not mirrored in terms of trastuzumab's therapeutic response. This is probably due to the fact that the effect of NAC on MUC4 positive tumors increased over time. Accordingly, at dissection, after 60 days of NAC supplementation, the difference in ^89^Zr-Trastuzumab uptake between the two groups was twice the one observed at baseline.

PET imaging with ^18^F-FDG, a radioactive analog of glucose, was also implemented. Although all methods of therapy monitoring were concordant, the unequivocal discrimination observed between treatment arms was more pronounced when monitored by ^18^F-FDG-PET. A 6-fold lesser accumulation of ^18^F-FDG was found in both trastuzumab groups for SKBR3 and only in the trastuzumab-NAC arm for JIMT1 tumors when compared to control. Also, a significant correlation was found between ^18^F-FDG uptake and the number of Ki67 positive cells (R^2^ = 0.79, *p* < 0.05, data not shown), supporting ^18^F-FDG-PET as a good surrogate for tumor proliferation activity in ductal breast carcinoma [28], corresponding to the BC cells in our model. Unfortunately an absolute decrease in SUV_max_ after therapy could not be demonstrated. This was most likely due to tumor growth (up to 40%) in the interval between the baseline ^18^F-FDG-PET and the start of the trastuzumab to allow the ^89^Zr-Trastuzumab PET examination. This issue was also previously described in clinical setting by our group [29].

The efficacy of trastuzumab treatment results mostly from the inhibition of the phosphorylation of HER2, hence and foremost the downstream PI3K-Akt pathway [6], which plays a critical role in growth factor-induced cell proliferation and survival [30]. Accordingly in our study, Akt phosphorylation was found significantly decreased in tumors responding to trastuzumab (alone for SKBr3 or only in combination with NAC for JIMT1).

The clinical importance of the Ki67 value in HER2 positive BC has previously been investigated. Studies showed that a Ki67 value ≤20% was predictive of pathological complete response [31] and associated with significantly higher post-recurrence survival [32]. In our study, the Ki67 proliferation index was significantly lowered in both trastuzumab treatment groups for SKBR3 and only in the trastuzumab-NAC arm for JIMT1 tumors, dropping to a value below 15 %. Trastzumab has also been reported to exerts its effect through HER2 downregulation, this effect was not demonstrated here most likely due to heterogeneity in HER2 staining [33].

HER2 immunostaining in our study was done using a clinically available and FDA approved IHC kit with a mAb recognizing the intracellular domain of HER2 (ICD) [26]; whereas immunoPET (and treatment) where based on trastuzumab raised against the extracellular domain (i.e. ECD IV). This epitope difference could explain the non-concordance found between HER2 IHC and ^89^Zr-Trastuzumab immunoPET in regards to the level of MUC4 as well as the therapeutic effect of trastuzumab.

In the present study we found an inverse correlation between MUC4 expression and ^89^Zr-Trastuzumab immunoPET in contrast with a positive correlation with HER2 IHC. Although seemingly contradictory, ^89^Zr-Trastuzumab findings are concordant with MUC4 hindering the accessibility of HER2-ECD henceforth the binding of (radiolabeled) trastuzumab [14, 19], while the HER2 IHC results are agreeing with MUC4 as modulator of the expression of HER2 [17, 18].

This further implies that tumors that would benefit of trastuzumab (or TDM1) are not necessarily the ones with the highest density of HER2, as they also might be the ones with the highest expression of MUC4. On the other hand, these tumors represent the ideal candidates for combining trastuzumab (or T-DM1) with NAC supplementation and as a result be the most favored by our proposed approach.

In addition, a significant correlation was found between ^89^Zr-Trastuzumab immunoPET (ECD) and the metabolic response after trastuzumab treatment assessed by ^18^F-FDG PET and not with HER2 expression (ICD).

This relationship between a specific HER2 domain and response to trastuzumab based treatment has also been demonstrated in clinical studies.

Recently, a clinical study on 180 HER2-positive patients under trastuzumab treatment explored the relationship between domain-specific HER2 expression of the tumor on IHC and the benefit of treatment for the patient in terms of disease-free survival (DFS). *Carvajal-Hausdorf et al*. revealed a differential benefit from trastuzumab therapy based on HER2 ECD expression rather than ICD in BC [35]. Similarly, our group demonstrated that ^89^Zr-Trastuzumab immunoPET (ECD) before treatment allowed the discrimination of patients benefiting from trastuzumab emtansine (T-DM1) with longer time to treatment failure, rather than their HER2 status (ICD/FISH) [29]. Both findings strongly advocate for a shift to an analysis of HER2 ECD rather than the currently used ICD when determining the eligibility of a patient for a targeted treatment against the ECD.

The present study is in concordance and further shows that increasing the amount of accessible HER2-ECD with NAC (on ^89^Zr-Trastuzumab), results in an increased therapeutic benefit of trastuzumab regardless of HER2-ICD (on IHC). Furthermore, our results imply that, despite an intact ICD expression, the benefit of trastuzumab-based treatment is greatly diluted when the tumor lesion presents with impaired accessibility to HER2 ECD. Henceforth, NAC supplementation could be of help in those circumstances. It could enhance the amount of available ECD without compromising the therapeutic effect of trastuzumab.

These issues of target accessibility are now even more critical with the advent of new antibody drug conjugates given as single agents, as certain mAbs might be inherently suboptimal for targeted drug delivery due to less accessible target ECD epitopes. Ergo, a possible course of action could be (1) opting for an mAb recognizing a more accessible membrane distal epitope, such as pertuzumab (ECD II [29]) which might represent a superior option for targeted drug delivery to HER2 rather than trastuzumab's ECD IV (eg. T-DM1) or (2) attempt to enhance the accessibility of the epitope for the mAb in question as proposed in this study.

The present study highlighted the potential role of molecular imaging with radiolabeled trastuzumab in gaining information regarding trastuzumab-HER2 interaction, in the investigation of a particular resistance mechanism and as a potential biomarker for response prediction. Although, our study focused on the already longstanding HER2-treatment trastuzumab, the same interactions with HER2 are expected for trastuzumab based treatments such as T-DM1. The latter is now widely offered and has in part substituted trastuzumab in the treatment of HER2 positive BC patients. Nonetheless, up to this day, T-DM1 administration is still preceeded by trastuzumab and also follows when resistance to the former arises. Hence, ways to possibly overcome resistance to trastuzumab remain highly relevant.

Taken together, our results demonstrate that NAC supplementation can significantly break MUC4-related resistance to trastuzumab by restoring HER2 hampered accessibility. The effect was revealed on ^89^Zr-Trastuzumab HER2-immunoPET, monitored by ^18^F-FDG-PET/CT and tumor volume measurements, and validated at the molecular level. Tentatively, this safe and promising approach may be offered to a subset of patients with HER2-positive BC tumors overexpressing mucins.

## MATERIALS AND METHODS

### ^89^Zr-labeling of trastuzumab

Labeling of trastuzumab and quality controls were performed as previously described [19]: 2.5mg of GMP grade N-SucDF-trastuzumab (VU Windesheim-VU Medisch Centrum, Amsterdam, Holland), synthesized by coupling desferrioxamine to trastuzumab, was radiolabeled with 111MBq ^89^Zr-oxalate (t _1/2_=78.4 h; Perkin Elmer). The specific activity was adjusted to 3.7MBq/100μg/100μL by adding unlabeled trastuzumab.

### Measurement of ^89^Zr radioactivity

^89^Zr-Trastuzumab accumulated activity was measured in a gamma-counter (Wallac Wizard, Perkin-Elmer) with background- and decay-correction as well as reference activity standards of 10%, 20% and 30% of the administered radioactivity.

### Cells

The human BC HER2-positive and MUC4-negative SKBr3 (ATCC) and HER2-positive and MUC4-positive JIMT1 (DSMZ) were cultured in DMEM high glucose (Lonza), supplemented with heat inactivated 10% FBS (Fisher) and antibiotics (penicillin-G (Sigma), streptomycin sulphate and kanamycine (both from Gentaur)) at 37°C and 5% CO_2_ in a fully humidified atmosphere. Cells were subcultured twice a week.

Cells were regularly checked for mycoplasma contamination using MycoAlert^®^ Mycoplasma Detection Kit (Lonza).

### Mouse model

Six-week-old athymic nu/nu female mice (Charles River) were inoculated subcutaneously on the right posterior leg with 3 × 10^6^ SKBr3 cells and on the left with 1.5 × 10^6^ JIMT1 cells suspended in 100μL of DMEM and Basement Membrane Extract (1:1; Cultrex, Trevigen). Imaging and treatments (Figure 1) were initiated two weeks after inoculation when tumors reached approximately 0.5 cm^3^. Mice were randomized to four different groups: (1) the CS group receiving sweetened drinking water and i.p. saline injections (*n* = 10), (2) the CT group receiving sweetened drinking water and trastuzumab injections (i.p., 5mg/kg [37, 38]) (*n* = 10), (3) the NS group receiving sweetened drinking water supplemented with NAC and saline i.p. injections (*n* = 9), and (4) the NT group receiving sweetened drinking water supplemented with NAC and trastuzumab injections (i.p., 5 mg/kg) (*n* = 10).

The drinking bottles were sweetened and refreshed twice a week until sacrifice for all groups. The drinking water of group NS and NT was supplemented with 4g/L NAC (Sigma Aldrich) and adjusted to pH 6.5–7.5 with sodium hydroxide [39, 40]. NAC supplemented in this manner resulted in a mean dose of 1 g of NAC per kg body weight per day [39].

Mice were observed daily for signs of discomfort or mortality throughout the treatment period and body weight was recorded weekly. All animal experiments were reviewed and approved by the Committee on Animal Ethics and Well Being of the Université Libre de Bruxelles (protocol 495N).

### Tumor growth

Tumor growth was monitored weekly by caliper measurements until sacrifice. Tumor volume was calculated using formula $\frac{\pi }{6}\times \sqrt[{2}]{{\left(LW\right)}^{3}}$ and later compared to the volume determined on computed tomography images.

### Small-animal PET-CT imaging

Studies were conducted using a μPET-CT (nanoScan^®^PET/CT, Mediso) on dual-tumor-bearing mice (*n* = 9–10/group). Each mouse was scanned three times, with an ^18^F-FDG and ^89^Zr-Trastuzumab PET-CT before treatment and an ^18^F-FDG PET-CT post treatment (Figure 1). The PET tracer (^18^F-FDG = 4.14 ± 0.36 MBq; ^89^Zr-Trastuzumab = 3.15 ± 0.24 MBq/100 μL) was injected in a lateral tail vein. Images were recorded for ^18^F-FDG at 1h and ^89^Zr-Trastuzumab 6 days after injection respectively. Two animals were scanned simultaneously under isoflurane anesthesia (induction: 3% isoflurane, 3L O_2_; maintenance: 1.5% isoflurane, 1.5L O_2_) and at 37°C using a thermoregulation unit (Minerve).

Baseline ^18^F-FDG PET showed homogenous and similar radiotracer uptake in all tumors and in all mice. The subsequent randomization was well balanced, with similar tumor ^18^F-FDG uptakes for the different arms prior to treatment in both types (SUV_max_: SKBR3: CS=0.61 ± 0.06, CT = 0.62 ± 0.06, NS = 0.60 ± 0.05 and NT = 0.64 ± 0.08; JIMT1: CS = 0.69 ± 0.07, CT = 0.56 ± 0.04, NS = 0.66 ± 0.06 and NT = 0.70 ± 0.07).

### *Ex-vivo* body distribution

After the last scan, at 6 days post ^89^Zr-Trastuzumab administration, animals were euthanized by cervical dislocation under anesthesia and an ex-vivo body distribution study was performed. ^89^Zr-Trastuzumab activity was measured in 16 organs (including tumors). Tissue uptake is presented as organ to blood ratio.

### Immunostaining

After dissection, tumors were immediately fixed in 10% formalin until the ex-vivo study was performed and radioactivity decayed. Samples were then transferred to 70% ethanol and stored at 4°C. Samples were processed and embedded in paraffin, and 4-μm sections were prepared for HE-staining. Tumor sections from a subset of animals from each cohort were also analyzed using immunostaining with different antibodies: anti-HER2 (4B5, Roche), anti-MUC4 (8G-7, Biocare Medical) anti-pAkt (D9E, Santa Cruz) and Ki67 (MIB-1, Dako) and subsequently stained with diaminobensidine (DAB, iview DAB detection kit, Ventana) except for pAkt staining where alkaline phosphatase red (ultraView Universal Alkaline Phosphatase Red, Ventana) was used.

Stained sections were imaged using NDP Slice Scanner (Hamamatsu). Three regions were selected at random on different part of the section and analyzed at ×20 magnification, using ImmunoMembrane and ImmunoRatio web applications. In brief, ImmunoMembrane software segments stained membrane regions from the user-submitted sample image and classifies the image into 0/1+,2+, or 3+ and calculates IM scores based on the membrane staining completeness and intensity; whereas, ImmunoRatio software segments DAB-stained and HE-stained nuclei regions from the user-submitted image and calculates the percentage of DABstained nuclear area over total nuclear area. Both applications generate a pseudo-colored overlay images matching the area segmentation [41].

### PET-CT image analysis

PET-CT image analysis and quantification were performed using PMOD software (PMOD Technologies, Switzerland). To define the volumes of interest (VOIs), tumors were manually contoured on the CT image. To quantify radioactivity within the tumor corresponding PET uptake values were normalized to the injected radioactivity per body weight (SUV).

The results are represented as the tumor volume for CT and the maximum uptake value of the VOI (SUV_max_) for PET. Changes in ^18^F-FDG uptake after tumor growth under the different treatment regimens are represented as the percentage difference between SUV_max_ post-treatment and SUV_max_ pre-treatment (%ΔSUV_max_). Lower %ΔSUV_max_ denoted lower tumor glucose metabolism in comparisons between treatment regimens.

### Statistics

Statistical analysis was done using ANOVA for the 4 animal group differences, student-T test when comparing two data replicates, extra sum-of-squares F test for curve differences, Pearson test for correlation. Statistics and graphs were performed using Graphpad Prism (GraphPad Software, San Diego, CA, USA). Data are presented as mean ± standard error of the mean (SEM) unless stated otherwise.

## SUPPLEMENTARY MATERIALS FIGURES


