# Supplementary material for: N-Acetylcysteine breaks resistance to trastuzumab caused by MUC4 overexpression in human HER2 positive BC-bearing nude mice monitored by 89Zr-Trastuzumab and 18F-FDG PET imaging
Source: Oncotarget. 2017 Apr 10;8(34):56185–98. doi: 10.18632/oncotarget.17015 (PMC5593554; doi:10.18632/oncotarget.17015)
Supplement: Supplementary file 1 [file oncotarget-08-56185-s001.pdf]

N-Acetylcysteine breaks resistance to trastuzumab caused by MUC4 overexpression in human HER2 positive BC-bearing nude mice monitored by ^{89}Zr-Trastuzumab and ^{18}F-FDG PET imaging

Supplementary Materials

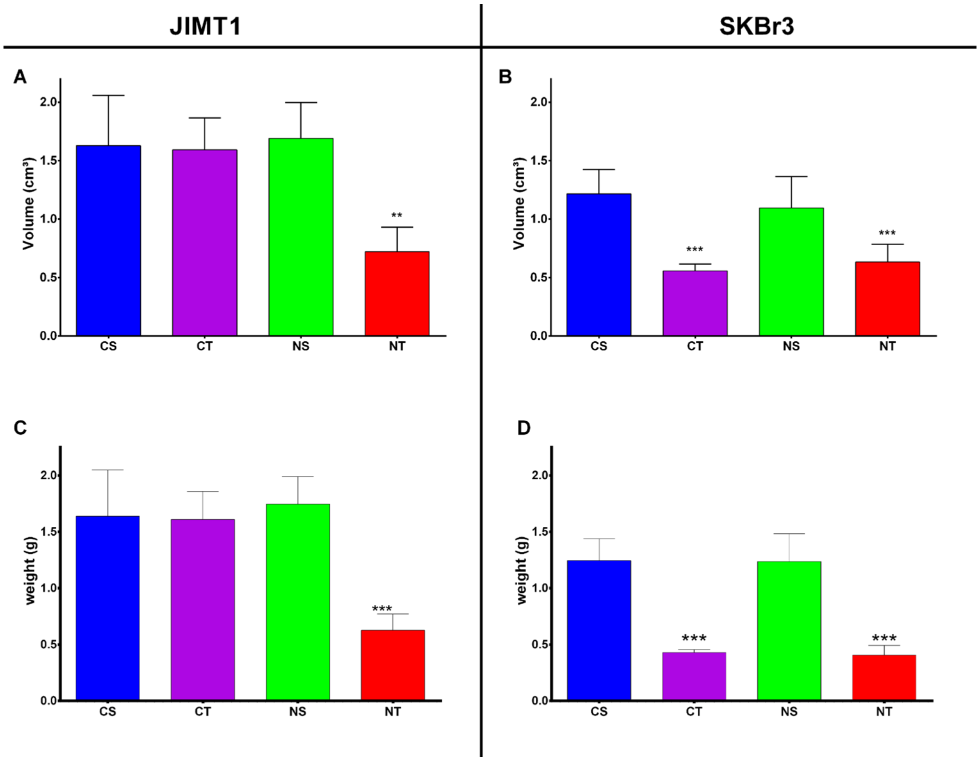

Supplementary Figure 1: The combination of trastuzumab with NAC supplementation results in smaller tumor volumes and weights of trastuzumab resistant tumors. The effect of the different treatment on JIMT1 (HER2+/MUC4+) and SKBr3 (HER2+/MUC4) tumors in dual-tumor-bearing mice randomized to the four treatment arms CS (Control+Saline; blue, n = 10), CT (Control+Trastuzumab; purple, n = 10), NS (NAC+Saline; green, n = 9) and NT (NAC+Trastuzumab; red n = 10) is represented per methodologies as follows: tumor volume of JIMT1 (**A**) and SKBr3 (**B**) tumors monitored by means of computed tomography images and tumor weight of JIMT1 (**C**) and SKBr3 (**D**) tumors at dissection. Data are represented as mean ± SEM, expressed in cm^3 for tumor volume and in grams (g) for tumor weight, with $^{**}p < 0.01$ and $^{***}p < 0.001$.

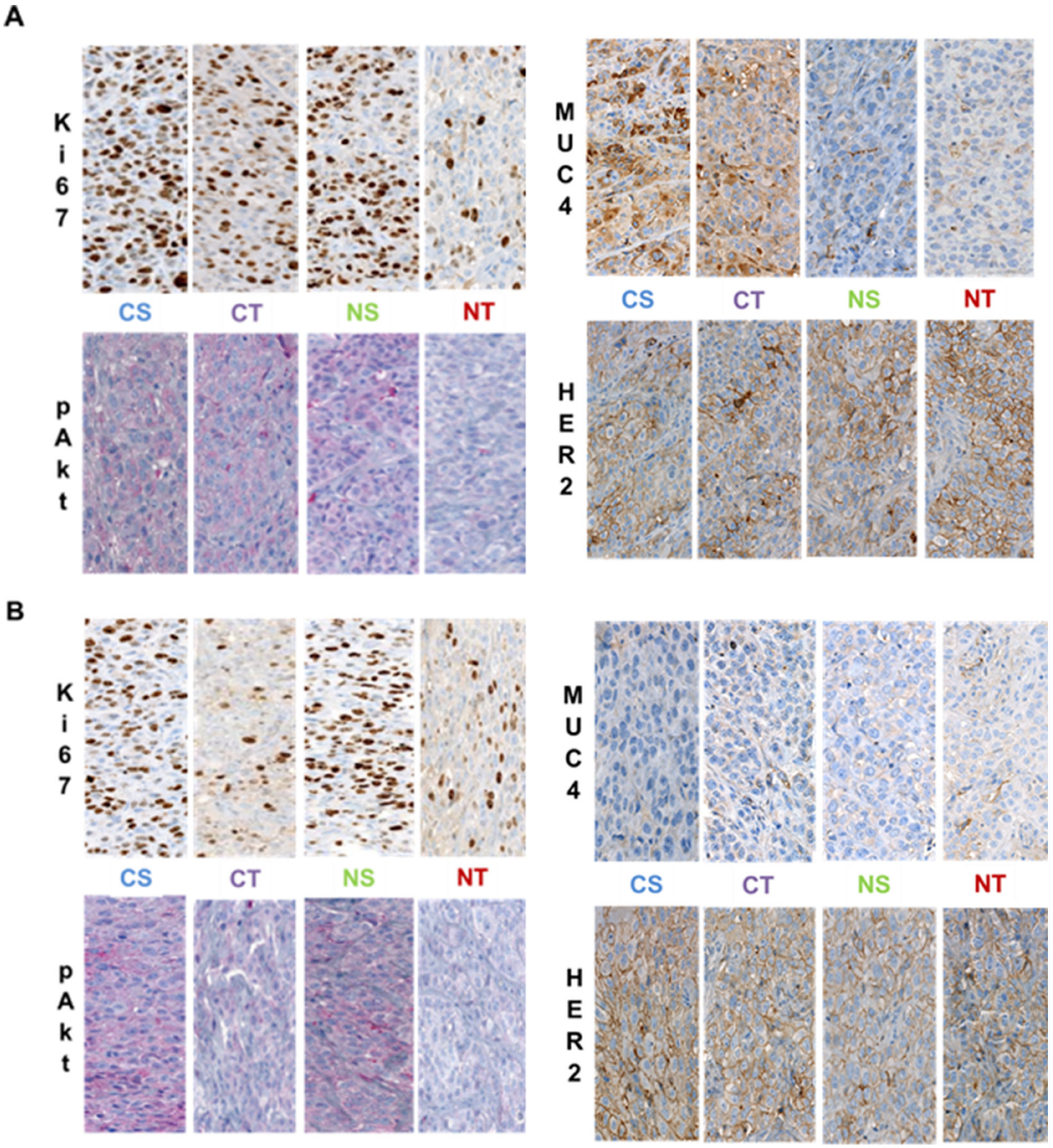

Supplementary Figure 2: Effect of combining trastuzumab with NAC supplementation on proliferation, the molecular Pi3K/Akt signaling pathway, MUC4 and HER2 expression in HER2 positive tumors. Representative FFPE stained sections of the pathological examination are shown as illustration for the proliferation index Ki67, pAkt , MUC4 and HER2 expression levels for (**A**) JIMT1 (HER2+/MUC4+) and (**B**) SKBr3 (HER2+/MUC4-) tumors in dual-tumor-bearing mice randomized to the four treatment arms CS (Control+Saline; blue, n = 5), CT (Control+Trastuzumab; purple, n = 5), NS (NAC+Saline; green, n = 5) and NT (NAC+Trastuzumab; red n = 5).
